# Fermentation of increasing ratios of grain starch and straw fiber: effects on hydrogen allocation and methanogenesis through *in vitro* ruminal batch culture

**DOI:** 10.7717/peerj.15050

**Published:** 2023-04-14

**Authors:** Siyu Yi, Xiumin Zhang, Xuezong Chen, Juwang Zhou, Cheng Gao, Zhiyuan Ma, Rong Wang, Zhiliang Tan, Min Wang

**Affiliations:** 1 Key Laboratory for Agro-Ecological Processes in Subtropical Region, Institute of Subtropical Agriculture, The Chinese Academy of Sciences, Changsha, China; 2 College of Animal Science and Technology, Guangxi University, Nanning, China

**Keywords:** Starch, Fiber, Rumen fermentation, Hydrogen, Methane

## Abstract

Grain starch has a faster rate of rumen fermentation than straw fiber and causes a rapid increase in ruminal molecular hydrogen (H_2_) partial pressure, which may promote other H_2_ sinks to compete H_2_ away from methanogenesis. The study was designed to investigate the effects of increasing ratios of grain starch to straw fiber on hydrogen allocation and methanogenesis through *in vitro* ruminal batch incubation. Corn grain and corn straw were employed as starch and fiber source respectively. Seven treatments were the ratios of corn grain to corn straw (RGS) being 0:6, 1:5, 2:4, 3:3, 4:2, 5:1, and 6:0. Elevating RGS increased dry matter (DM) degradation and decreased methane (CH_4_) and hydrogen gas (gH_2_) production relative to DM degraded. Elevating RGS increased volatile fatty acid (VFA) concentration, propionate molar percentage and microbial protein (MCP) concentration, decreased acetate molar percentage, acetate to propionate ratio and estimated net metabolic hydrogen ([H]) production relative to DM degraded. Elevating RGS decreased the molar percentage of [H] utilized for CH_4_ and gH_2_ production. In summary, increasing ratios of grain starch to straw fiber altered rumen fermentation pathway from acetate to propionate production, reduced the efficiency of [H] production with the enhancement of MCP synthesis, and led to a reduction in the efficiency of CH_4_ and gH_2_ production.

## Introduction

Methane (CH_4_) is an important greenhouse gas, which is the second largest global radiation driver after carbon dioxide (CO_2_), whereas its global warming potential is about 28 times higher than CO_2_ (Finn, Dalal & Klieve, 2015; Zubieta et al., 2021). Thus, CH_4_ has received great attention worldwide for its impact on global climatic change. Globally, enteric CH_4_ emissions makes up about 87-97 Tg CH_4_ per year (Chang et al., 2019), and contributes to an important source of global anthropogenic greenhouse gas emission (Wang et al., 2022). Furthermore, CH_4_ emissions represent 2–12% of dietary gross energy and is strongly associated with efficiency of ruminants production (Johnson & Johnson, 1995; Knapp et al., 2014). Therefore, CH_4_ mitigation is beneficial to the environment and animal performance.

Molecular hydrogen (H_2_) is a precursor of ruminal methanogenesis and is mainly produced during the fermentation of carbohydrates to volatile fatty acid (VFA) (Janssen, 2010). Other H_2_ sinks, such as reductive acetogenesis, biohydrogenation, propionate production and microbial protein (MCP) synthesis (Lan & Yang, 2019) serve as metabolic hydrogen ([H]) competitors in the rumen microbial ecosystem (Ellis et al., 2008). Increasing concentrate ratio represents an effective dietary strategy to reduce enteric methane (CH_4_) emissions in ruminants. In comparison to forage fiber, starch has a faster rate of rumen fermentation and ATP production and is always accompanied with a rapid increase in ruminal H_2_ partial pressure (Wang et al., 2016a; Xu et al., 2018). Such increasing H_2_ partial pressure are always associated with enhanced competition of H_2_ utilization for H_2_ sinks other than methanogenesis, which needs further investigation.

We hypothesized that elevating the ratios of corn grain to corn straw (RGS) could decrease the contribution of H_2_ utilization for methanogenesis, thus leading to the enhancement of other [H] utilization pathways, such as propionate production and MCP synthesis. *In vitro* ruminal batch culture was employed, as it is effective method to measure the actual net fermentation products. Increasing ratios of grain starch to straw fiber was then achieved by replacing corn straw with corn grain. We measured the kinetics of total gas, CH_4_ and H_2_ gas (gH_2_) productions, fermentation end products, estimated net [H] production and MCP concentration after 48-h *in vitro* ruminal fermentation. Our results demonstrate that increasing the level of starch to fiber reduced intensity of CH_4_ production by altering fermentation pathway and diverting H_2_ into alternative sinks.

## Materials and Methods

### Research ethics

This study was conducted at Institute of Subtropical Agriculture, the Chinese Academy of Sciences, Changsha, China. The procedures of animal experiments were carried out in accordance with the Animal Care and Use Committee of the Institute of Subtropical Agriculture, Chinese Academy of Sciences, Changsha, China (Approval NO. ISA 2020-0019).

### Experimental design

Corn grain and corn straw (Table 1) were served as starch and fiber source, respectively. The seven treatments that were examined differed in the RGS and included 0:6, 1:5, 2:4, 3:3, 4:2, 5:1, and 6:0. The experiment was conducted by a completely randomized block design, which included three runs with each treatment containing four fermentation bottles (replicates). Samples were ground to pass through a 1-mm aperture sieve (Daoxujian Instruments Co. Ltd., Shaoxing, China).

**Table 1:** Chemical compositions of corn grain and corn straw (expressed in g/kg of DM).

Item	Corn grain	Corn straw
OM	985	915
CP	76.4	38.7
NDF	132	792
ADF	34.5	482
Starch	747	72.4
GE (MJ/kg DM)	16.5	14.8

**Notes.**

ADF acid detergent fiber CP crude protein GE gross energy NDF neutral detergent fiber OM organic matter

### *In vitro* ruminal batch incubation

Rumen fluid was collected from two of three adult male Xiangdong black goats (BW 30.0 ±1.50 kg) with permanent rumen cannula before morning feeding. The three goats were in healthy state and rumen fluid samples were taken until the end of the experiment. The goats were fed a total mixed diet containing corn straw and concentrated mixture (1:1) with the crude protein (CP) content of 137 g/kg dry matter (DM) and the content of neutral detergent fiber (NDF) 380 g/kg DM, free to drink water, and the goat houses are ventilated. The rumen fluid was filtered through four layers of cheesecloth into a pre-warmed insulated bottle and taken to the laboratory.

Approximately 0.6 g of substrate was accurately weighed into a 135-mL fermentation bottle. Then buffered rumen fluid containing 12 mL of rumen fluid and 48 mL of McDougall’s buffer (Cone & Becker, 2012) were added into bottle under a stream of CO_2_ at 39.5 °C. Bottles were immediately placed into the automatic incubation system described by Wang et al. (2016b), with venting pressure set at 10.0 kPa. As the incubation bottle was in line with gas chromatograph (GC, Agilent 7890 A, Agilent, Palo Alto, California, USA) *via* a computer-controlled three way solenoid valve, the released gas was automatically vented into a GC for measuring CH_4_ and gH_2_ concentrations. Gas production (GP), CH_4_ and gH_2_ accumulations were calculated using the equation described by Wang et al. (2013a).

*In vitro* ruminal fermentation was stopped at 48 h. About 2 mL of liquid without visible particles were collected from each bottle and centrifuged at 15, 000 *g* for 10 min at 4 °C. The supernatant (1.5 mL) was acidified by 0.15 mL of 25% (w/v) metaphosphoric acid, and stored at −20 °C for analysis of VFA and ammonia-N. The pH was measured immediately with a portable pH meter (Starter 300; Ohaus Instruments Co. Ltd., Shanghai, China). Approximately eight mL of samples were collected for measuring MCP after intense shaking of the bottle to ensure that representative portions of liquid and particle fractions. Solid residues were filtered into pre-weighed Gooch filter crucibles, dried at 105 °C to constant weight and weighed to determine degradation of incubated substrates.

Two bottles in each run were used for measuring pH and DM degradation, and the other two bottles were used for obtaining samples for measuring fermentation end-product and MCP. Each run was repeated three times, each on different days, so that each treatment was conducted in triplicate.

### Sample analyses

The DM content was determined by drying at 105 °C for 24 h in an oven, and the organic matter (OM) content was determined by ashing at 550 °C for 12 h in a muffle furnace. Gross energy (GE) was measured using an isothermal automatic calorimeter (5EAC8018; Changsha Kaiyuan Instruments Co. Ltd, Changsha, China). The contents of CP (N ×6.25) in feed samples were determined according to procedures of AOAC (1995). The contents of NDF and acid detergent fiber (ADF) in feed samples were determined according to the methods described by Van Soest, Robertson & Lewis (1991) and expressed as inclusive of ash. Heat stable *α*-amylase was added to for NDF analysis. The starch content was determined after pre-extraction with 80% ethanol (*v*/*v*), and glucose released from starch by enzyme hydrolysis was measured using amyloglucosidase (Sigma) according to Karthner & Theurer (1981).

Volatile fatty acid concentration was measured according to the procedure described by Wang et al. (2014), using a GC (Agilent 7890 A, Agilent Inc., Palo Alto, California, USA). Ammonia-N concentration was measured colorimetrically according to Chaney & Marbach (1962). Rumen microorganisms was separated from feed particles according to Makkar et al. (1982) with filtration (four layers of gauze), shaking (125 rpm/min for 1h) and centrifugation (150 × *g* for 10 min), and microbial nitrogen production was measured by Microplate Reader (Infinite M200 PRO\spark, Tecan Inc., Männedorf, Switzerland) according to Bradford (1976), Using a Coomassie brilliant blue kit (Build a biopharmaceutical research institute, Nanjing, China).

### Calculations and statistical analysis

The kinetics of total gas and CH_4_ analyzed using the equation provided by Wang, Tang & Tan (2011), which was expressed as follows:

\begin{eqnarray*}{GP}_{t}=VF \frac{1-exp(-kt)}{1+exp(b-kt)} \end{eqnarray*}

where *GPt* is the accumulated gas/CH_4_ production at time t (mL/g); *VF* is the final asymptotic gas/CH_4_ volume (mL/g); *k* is the fractional rate of gas/CH_4_ production (/h); *b* is the shape parameter of gas/CH_4_.

The kinetics of gH_2_ production was analyzed using the equation provided by Wang et al. (2013b), which was expressed as follows:

\begin{eqnarray*}{V}_{H2t}= \frac{V{F}_{H2} \left\{ 1-\mathit{exp} \left[ -{k}_{H2} \left( t-la{g}_{H2} \right) \right] \right\} \{ 1+{c}_{H2}\mathit{exp} \left[ -{\mu }_{H2} \left( t-la{g}_{H2} \right) \right] \} }{1+exp[{b}_{H2}-{k}_{H2} \left( t-la{g}_{H2} \right) ]} \end{eqnarray*}

where *V*_*H*2*t*_ is the accumulated gH_2_ production at time t (mL/g); *VF*_*H*2_ is the final asymptotic gH_2_ volume (mL/g), *b*_*H*2_ and *c*_*H*2_ are shape parameters of gH_2_ curve without dimension, *k*_*H*2_ is the fractional rate of gH_2_ production (/h), µ_*H*2_ is the fractional rate of gH_2_ utilization (/h), and *lag*_*H*2_ is discrete lag time (h).

The stoichiometric equations developed by Wang et al. (2014) was used to calculate the estimated net [H] production (P_NH2_, mM) and estimated [H] production relative to the amount of total VFA produced (R_NH2_, moL/100 mol of VFA), which was expressed as follows:

P_NH2_ = 2(Ace + But + Isobut) − (Pro + Val + Isoval)

R_NH2_ = 100 P_NH2_/(Ace + But + Isobut + Pro + Val + Isoval)

where ace, but, pro, val, isobut and isoval were concentration (mM) of acetate, propionate, valerate, isobutyrate, and isovalerate respectively.

*In vitro* DM degradation (DMD) was calculated using the equation provided by Zhang et al. (2018), which was expressed as follows:

\begin{eqnarray*}DMD~(g/kgofDM)=[1-{W}_{1}\times ({V}_{1}/{V}_{2})/{W}_{2}]\times 1,000 \end{eqnarray*}

where W_1_ is the DM weight of the residue after 48 h of incubation; W_2_ is the DM weight of substrate before incubation; V_1_ is the volume of buffered rumen fluid in the bottle before sampling (*i.e.,* 60 mL); V_2_ is the volume of buffered rumen fluid in the bottle after sampling (*i.e.,* 56 mL).

The data were analyzed using general linear model (GLM) with SPSS 26.0 (Chicago, IL, USA), and are presented as mean and SEM. The analytic model included treatment (*n* = 7) as fixed effect and run (*n* = 3) as random effect, and were analyzed for linear or quadratic responses to ratios of corn grain to corn straw using orthogonal contrasts. Statistical significance was considered at *P* ≤ 0.05 with 0.05 < *P* ≤ 0.10 considered as a tendency.

## Results

### Impacts of the ratios of corn grain to corn straw (RGS) on gas production

**Figure 1:**
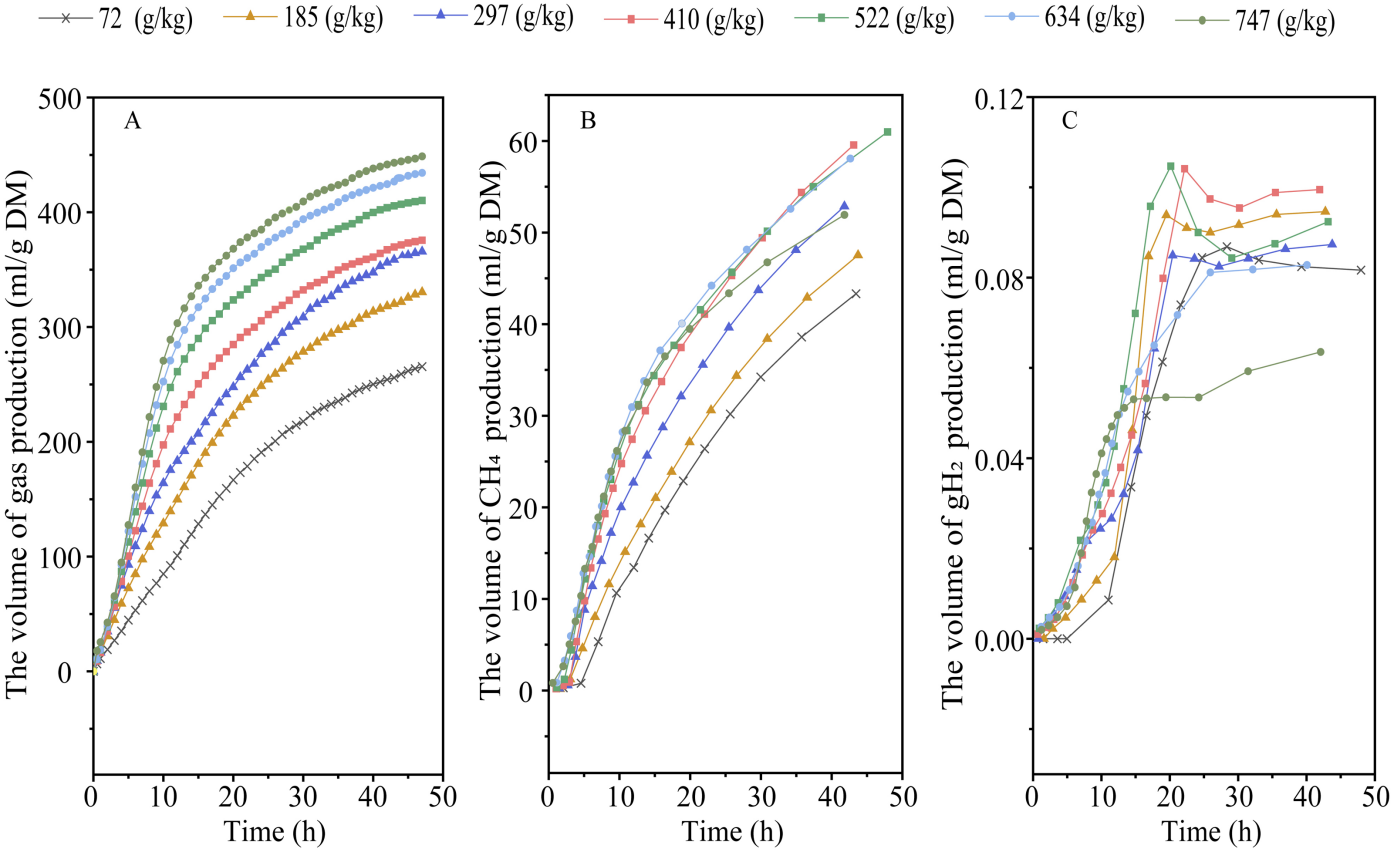
Effect of increasing the ratios of corn grain to corn straw (RGS) on total gas (A), methane (CH_4_, B) and hydrogen gas (gH_2_, C) production through 48-h *in vitro* ruminal fermentation.

Elevating RGS increased DMD (*P*_*linear*_ < 0.001) and altered the kinetic of gas production, with an increase in 48-h gas production, final asymptotic gas production, and fractional rate of gas production (*P*_*linear*_ < 0.001; *P*_*quadratic*_ < 0.01) (Fig. 1A and Table 2). Increasing RGS altered the kinetics of CH_4_ accumulation, with an increase in 48-h CH_4_ production (*P*_*linear*_ and *P*_*quadratic*_ < 0.001), final asymptotic CH_4_ production (*P*_*quadratic*_ < 0.001), and fractional rate of CH_4_ production (*P*_*linear*_ < 0.001; *P*_*quadratic*_ = 0.007), and a reduction in 48-h CH_4_ production relative to DM degraded (*P*_*linear*_ and *P*_*quadratic*_ < 0.001) (Fig. 1B and Table 3). With the increase of RGS, the 48-h gH_2_ production (*P*_*linear*_ = 0.011; *P*_*quadratic*_ = 0.003), final asymptotic gH_2_ production (*P*_*linear*_ = 0.03; *P*_*quadratic*_ = 0.002), and the 48-h gH_2_ production relative to DM degraded (*P*_*linear*_ < 0.001) were decreased, whereas the fractional rate of gH_2_ utilization was increased (*P*_*linear*_ = 0.035) (Fig. 1C and Table 3).

### Impacts of the ratios of corn grain to corn straw (RGS) on rumen fermentation

Elevating RGS decreased pH (*P*_*linear*_ < 0.001) and increased total VFA concentration (*P*_*linear*_ < 0.001). Improving RGS decreased acetate (*P*_*linear*_ < 0.001), butyrate (*P*_*quadratic*_ = 0.002), isobutyrate (*P*_*quadratic*_ = 0.02) molar percentage and acetate to propionate ratio (*P*_*linear*_ < 0.001). Improving RGS increased propionate, valerate and isovalerate molar percentage (*P*_*linear*_ < 0.001; *P*_*quadratic*_ < 0.01) (Table 4). With the increase of RGS, the estimated net [H] production (*P*_*linear*_ < 0.001; *P*_*quadratic*_ = 0.005) increased, whereas the estimated net [H] production relative to DM degraded (*P*_*linear*_ < 0.001) and total VFA produced (*P*_*linear*_ < 0.001; *P*_*quadratic*_ = 0.004) were decreased. Increasing RGS decreased molar percentage of [H] utilized for CH_4_ (*P*_*quadratic*_ = 0.003) and gH_2_ (*P*_*linear*_ < 0.001) production. Furthermore, the MCP (*P*_*linear*_ < 0.001; *P*_*quadratic*_ < 0.001) and ammonia-N concentrations (*P*_*quadratic*_ = 0.004) increased with the increase in RGS (Table 5).

## Discussion

It is well known that corn grain and corn straw have different types of carbohydrate components and are rich in starch and fiber, respectively. Fermentation rate of carbohydrate depends on their monosaccharide and bond composition, molecular size, sugar arrangement at molecular level and physical morphology (Gidley, 2013; Wang et al., 2019a). Starch is mainly formed by *α*-1,4 glycosidic bond and easily hydrolyzed by enzymes (Kim et al., 2017; Xu et al., 2018). The plant cell wall is composed of lignocellulose which contains cellulose, hemicellulose, pectin and lignin (Thakur & Thakur, 2016). The cellulose is made up of *β*-1,4 linked glucose molecules and cellulose polymers form crystalline structures, which has acid and enzymatic hydrolysis resistances (Thakur & Thakur, 2016). Compared with starch, cellulose and hemicellulose are also less susceptible to microbial degradation in the rumen (Zhang et al., 2017; Dias et al., 2018). In our study, elevating RGS linearly increased substrate degradation, gas production and the fractional rate of gas production, indicating a greater and faster rumen degradability.

Methane is one of the end-products generated during ruminal carbohydrate fermentation (Wang et al., 2019b). The amount of CH_4_ produced is related to the degree of substrate degradation and efficiency of CH_4_ production (*i.e.,* CH_4_ produced per unit of substrate degraded) (Janssen, 2010). It is not surprising that elevated RGS linearly increased 48-h and final asymptotic volume of CH_4_ production, and fractional rate of CH_4_ production, as starch had greater rate and extent of fermentation than straw fiber. However, the efficiency of CH_4_ production varies during the fermentation of starch and fiber. Increasing dietary starch content has been widely reported to reduce intensity of enteric CH_4_ emissions (Wang et al., 2016a; Bougouin et al., 2018). In the present study, elevating RGS linearly decreased amount of CH_4_ produced per unit of DM degraded, indicating a reduction in the efficiency of CH_4_ production.

**Table 2:** Effects of increasing the ratios of corn grain to corn straw (RGS) on dry matter degradation (DMD) and the kinetic parameters of gas production (GP) after 48-h *in vitro* ruminal incubation.

Items	RGS							SEM	*P*-value	
	0:6	1:5	2:4	3:3	4:2	5:1	6:0		*Linear*	*Quadratic*
DMD (g/kg)	511	579	645	706	777	854	916	30.5	<0.001	0.592
GP (mL/g of DM)	270	305	360	384	412	428	444	13.6	<0.001	<0.001
V*F*_GP_ (mL/g of DM)	307	338	384	397	411	418	432	9.8	<0.001	0.007
k_GP_ (10^−2^/h)	5.43	5.22	5.84	6.97	9.65	12.81	16.08	0.94	<0.001	0.005

**Notes.**

DMD dry matter degradation GP gas production k_GP_ the fractional rate of gas production V*F*_GP_ the final asymptotic volume of total gas production

**Table 3:** Effects of increasing the ratios of corn grain to corn straw (RGS) on the kinetic parameters of methane (CH_4_) and hydrogen gas (gH_2_) production after 48-h *in vitro* ruminal incubation.

Items	RGS							SEM	*P*-value	
	0:6	1:5	2:4	3:3	4:2	5:1	6:0		*Linear*	*Quadratic*
CH_4_										
mL/g of DM	44.8	49.4	53.4	57.7	58.9	57.7	52.5	1.26	<0.001	<0.001
mL/g of DDM	88.0	85.6	82.9	81.7	75.8	67.6	57.3	2.58	<0.001	<0.001
V*F*_CH4_ (mL/g of DM)	46.1	53.4	55.2	56.2	56.0	51.9	46.8	1.17	0.970	<0.001
k_CH4_ (10^−2^/h)	8.40	6.70	7.21	8.48	9.81	12.76	16.54	0.871	<0.001	0.007
gH_2_										
mL/g of DM	0.097	0.110	0.102	0.108	0.102	0.098	0.076	0.005	0.011	0.003
mL/g of DDM	0.19	0.19	0.16	0.15	0.13	0.12	0.08	0.011	<0.001	0.344
V*F*_H2_ (ml/g of DM)	0.095	0.110	0.103	0.110	0.104	0.100	0.076	0.005	0.030	0.002
k_H2_ (10^−2^/h)	34.0	43.3	30.3	26.7	27.1	28.5	33.9	2.51	0.281	0.252
µ_H2_ (h)	4.16	3.89	5.56	5.96	6.68	6.54	8.00	0.541	0.035	0.986

**Notes.**

DDM degraded dry matter k_CH4_ the fractional rate of CH_4_ production k_H2_ the fractional rate of gH_2_ production V*F*_CH4_ the final asymptotic volume of CH_4_ production V*F*_H2_ the final asymptotic volume of gH_2_ production µ_H2_ the fractional rate of gH_2_ utilization

**Table 4:** Effects of increasing the ratios of corn grain to corn straw (RGS) on ruminal pH and the profile of volatile fatty acids (VFA) after 48-h *in vitro* ruminal incubation.

Items	RGS							SEM	*P*-value	
	0:6	1:5	2:4	3:3	4:2	5:1	6:0		*Linear*	*Quadratic*
pH	6.58	6.53	6.49	6.44	6.40	6.34	6.27	0.026	<0.001	0.067
Total VFA (mM)	56.8	64.4	66.3	72.0	79.0	84.9	86.0	2.57	<0.001	0.490
Molar percentage of individual VFA (moL/100 moL)										
Acetate	71.0	69.0	68.1	66.3	64.4	62.4	60.4	0.80	<0.001	0.122
Propionate	19.8	20.9	21.5	22.9	24.6	27.0	29.6	0.75	<0.001	0.001
Butyrate	5.89	6.49	6.52	6.67	6.61	6.30	5.82	0.140	0.647	0.002
Isobutyrate	1.04	1.09	1.10	1.12	1.13	1.07	0.99	0.022	0.445	0.020
Valerate	0.86	0.97	1.04	1.12	1.20	1.22	1.26	0.033	<0.001	0.009
Isovalerate	1.37	1.60	1.75	1.92	2.04	2.02	1.93	0.059	<0.001	<0.001
Acetate to propionate ratio	3.61	3.30	3.17	2.89	2.62	2.32	2.04	0.119	<0.001	0.361

**Table 5:** Effects of increasing the ratios of corn grain to corn straw (RGS) on estimated net metabolic hydrogen ([H]), microbial protein (MCP) and ammonia-N concentrations after 48-h *in vitro* ruminal incubation.

Items	RGS							SEM	*P*-value	
	0:6	1:5	2:4	3:3	4:2	5:1	6:0		*Linear*	*Quadratic*
P_NH2_										
mM	76.1	83.5	84.2	87.9	92.0	92.9	87.4	1.90	<0.001	0.005
mM/g of DDM	149.7	144.9	130.8	124.3	118.3	108.8	95.4	4.77	<0.001	0.642
R_NH2_	134	130	127	122	116	109	102	2.4	<0.001	0.004
Molar perentage of [H] utilized (moL/100 moL P_NH2_)										
CH_4_	49.9	49.5	52.5	53.8	51.7	49.5	47.2	0.68	0.227	0.003
gH_2_	0.053	0.055	0.050	0.050	0.045	0.042	0.034	0.002	<0.001	0.076
Others	50.1	50.4	47.4	46.1	48.2	50.4	52.8	0.68	0.222	0.003
Ammonia-N (mM)	17.2	19.0	19.0	21.1	21.0	20.0	17.8	0.67	0.262	0.004
MCP (mg/mL)	0.75	0.73	0.78	0.81	0.84	0.91	1.08	0.026	<0.001	<0.001

**Notes.**

CH_4_ methane DDM degraded dry matter gH_2_ hydrogen gas MCP microbial protein P_NH2_ estimated net [H] production R_NH2_ estimated [H] production relative to the amount of total VFA produced

The fermentation of carbohydrates to VFAs results in the H_2_ production which is mainly consumed by methanogens to produce CH_4_ (Yi et al., 2022). The unused H_2_ will be evolved from liquid to gas phase and finally vent into air. Normally, the ruminal H_2_ partial pressure is very low to facilitate the rumen fermentation (Janssen, 2010). Rooke et al. (2014) report that H_2_ emissions account for less than 2% of the estimated total H_2_ produced during rumen fermentation. In our study, both 48-h and final asymptotic gH_2_ production was less than one mL/g DM, indicating that most of H_2_ produced were utilized. Furthermore, increasing RGS had different profile of gH_2_ production such as increased fractional rate of gH_2_ consumption. Elevating RGS showed quadratically reduction in the 48-h and final asymptotic volume of gH_2_ production and linearly reduction in the amount of gH_2_ produced per unit of DM degraded. We propose that corn grain starch exhibited lower efficiency of H_2_ production than corn straw fiber, which might be related to their different pathways of rumen fermentation.

Fermentation of feed rich in grain starch produces more propionate and butyrate, and less acetate than feed rich in cellulose and hemicellulose (Janssen, 2010; Bayat et al., 2017). In our study, elevating RGS linearly increased propionate molar percentage and estimated net [H] production, and linearly decreased acetate molar percentage and acetate to propionate ratio. Our results were consistent with previous *in vivo* studies, which found that elevating dietary starch increased propionate molar percentage and decreased the acetate molar percentage (Bayat et al., 2017; Dias et al., 2018). Formation of acetate and butyrate from carbohydrates results in net [H] production, whereas formation of propionate from pyruvate causes net [H] utilization (Ungerfeld, 2013). Our data showed that elevating RGS decreased net estimated [H] production per units of DM degraded and estimated [H] production relative to the amount of total VFA produced, although an increase in net estimated [H] production was observed. We also observed that less than 55% of the estimated net [H] produced was incorporated into CH_4_ and gH_2_, indicating that a significant amount of [H] was redirected into other fermentation products other than CH_4_ and gH_2_. Elevating RGS quadratically decreased proportion of [H] produced for CH_4_ production, indicating that elevated starch content may enhance the combination of [H] with other H_2_ sinks. Rapid grain starch fermentation causes a fast increase in H_2_ partial pressure (Wang et al., 2016a; Wang et al., 2019b), which may energetically promote other H_2_ utilization pathways.

Microbial protein is a alternative ruminal H_2_ sink, which synthesized by utilizing [H] and ammonia-N for synthesis of amino acids in the rumen (Lu et al., 2019). Microbial growth and protein synthesis requires utilization of ATP generated during rumen fermentation (Zhu et al., 2013; Xu et al., 2018). Non-fiber carbohydrates is the efficient energy substrate for ruminal microorganisms, and thus could promote the ammonia-N incorporation into MCP synthesis (Cantalapiedra-Hijar et al., 2014; Lu et al., 2019). In our study, elevating RGS linearly increased MCP concentration, leading to a quadratical change in ammonia-N concentration. Ammonia-N concentration is determined by the balance between its production and utilization, and thus related to the substrate degradation rate and MCP synthesis. Increasing RGS can result in a more rapidly available energy source for microorganisms which may promote the growth of ruminal microorganisms, and thus improve the utilization of ammonia-N and [H] for MCP synthesis (Calsamiglia et al., 2010; Zhang et al., 2017). Other studies also indicate that more [H] can be incorporated into microbial biomass when available H_2_ is increased (Ungerfeld, 2015; Lan & Yang, 2019). Thus, starch fermentation is beneficial to MCP synthesis in compared with straw fiber, which also contributed to the reduction in methanogensis in high starch treatments.

## Conclusions

Corn grain starch has faster and greater rumen degradability than corn straw fiber. Elevating the ratios of corn grain to corn straw decreased efficiency of CH_4_ and gH_2_ production, although it increased the CH_4_ production. Such reduction in efficiency of CH_4_ production can be caused by the shift of rumen fermentation pathways from acetate to propionate production with a reduction in efficiency of [H] production and increased MCP synthesis. Further researches are needed to investigate the mechanism of [H] transactions in the rumen ecosystem of ruminants fed with starchy *versus* fibrous diets.

##  Supplemental Information

10.7717/peerj.15050/supp-1 Supplemental Information 1 The original data for Table 2, Table 3, Table 4, Table 5, and Fig. 1

10.7717/peerj.15050/supp-2 Supplemental Information 2 Author Checklist

## Additional Information and Declarations

### Data Availability

The following information was supplied regarding data availability: The raw data are available in the Supplemental Files.
